# Computational approaches for interpreting scRNA‐seq data

**DOI:** 10.1002/1873-3468.12684

**Published:** 2017-06-12

**Authors:** Raghd Rostom, Valentine Svensson, Sarah A. Teichmann, Gozde Kar

**Affiliations:** ^1^ Wellcome Trust Sanger Institute Cambridge UK; ^2^ The European Bioinformatics Institute (EMBL‐EBI) Cambridge UK

**Keywords:** single‐cell analysis methods and tools, single‐cell genomics

## Abstract

The recent developments in high‐throughput single‐cell RNA sequencing technology (scRNA‐seq) have enabled the generation of vast amounts of transcriptomic data at cellular resolution. With these advances come new modes of data analysis, building on high‐dimensional data mining techniques. Here, we consider biological questions for which scRNA‐seq data is used, both at a cell and gene level, and describe tools available for these types of analyses. This is an exciting and rapidly evolving field, where clustering, pseudotime inference, branching inference and gene‐level analyses are particularly informative areas of computational analysis.

## Abbreviations

**Dpt**, diffusion pseudotime

**FACS**, fluorescence activated cell sorting

**GPLVM**, Gaussian Process Latent Variable Model

**IVT**, *in vitro* transcription

**LR**, likelihood ratio

**MDS**, multidimensional Scaling

**MST**, minimum spanning tree

**NB**, negative binomial

**PCA**, principal component analysis

**RT**, reverse transcription

**scLVM**, single‐cell latent variable model

**scRNA‐seq**, single‐cell RNA sequencing

**SNN**, shared nearest neighbour

**UMIs**, unique molecular identifiers

**WGCNA**, weighted gene coexpression network analysis

**ZIFA**, zero‐inflated factor analysis

While transcriptomic studies have, for many years, provided insight into mRNA expression and regulation, technological advances have allowed the quantification of transcripts at an unprecedented resolution. By sequencing the mRNA component of individual single cells, it has now become possible to study gene expression at an entirely new level, opening the door to novel biological questions which were not possible using population‐level RNA sequencing. For example, the variability in splicing 1, 2, 3, 4, 5 and allelic expression 3, 6, 7, 8 between cells has been shown, along with analysis of the stochastic gene expression and transcriptional kinetics 9, 10. Furthermore, single‐cell RNA‐sequencing (scRNA‐seq) data have allowed fine‐grained analysis of developmental trajectories 11, 12, 13 and identification of rare cell types 14, 15.

In order to obtain scRNA‐seq data, cells must first be isolated individually in an accurate and rapid manner. Initially, microscopic manipulation provided a reliable method to isolate single cells through physical separation using a capillary pipette, and may still play an important role in systems where few cells are available. However, the high labour and low‐throughput nature of this technique has resulted in it being surpassed in much current research by higher throughput methods. Fluorescence‐activated cell sorting (FACS) provides an efficient way to isolate a large number of cells in a rapid manner, and also allows the possibility of labelling cells with multiple fluorescent proteins. Size or marker selection is commonly used, and through ‘index sorting’, the data for each cell can be recorded as a reference in downstream analysis. Despite the prevalence of this method, the high number of starting cells required, along with the potential damage caused by the staining and physical stress of the process, means it may be a problematic approach. More recently, microfluidics have emerged as a key method for capturing single cells, allowing isolation in small volumes within a closed system, often followed directly by amplification and downstream reactions. The small volume in which these reactions occur increases the capture efficiency and lowers the reagent cost. Finally, techniques involving the isolation of single cells in microdroplets, such as DropSeq 16 and InDrop 17, have rapidly expanded the high‐throughput nature of scRNA‐seq – allowing processing of tens of thousands of cells in a short space of time. The small volume of reactions, once again, decreases the cost per cell. Over time, these methods will continue to increase in speed, efficiency and reliability, further improving throughput of single‐cell isolation.

Many protocols exist for the subsequent reverse transcription (RT), amplification, and library preparation prior to sequencing. Poly(T) priming is used to select polyadenylated mRNA for reverse transcription, however, only an estimated 10–20% of transcripts are sampled. This produces a lot of noise at the RT stage, and particularly affects lowly expressed genes 18. Methods then differ in their approach to second‐strand synthesis, either using poly(A) tailing, leading to a 3′ bias, or template‐switching to produce full‐transcript coverage. Amplification can be achieved through two methods: linear *in vitro* transcription (IVT) or exponential PCR, each with its own advantages and drawbacks. Ziegenhain *et al*. 19 and Svensson *et al*. 20 provide a comprehensive experimental and computational comparison of most of the protocols commonly used. Following cDNA amplification, library preparation is most commonly carried out using the commercially available Nextera kit and sequencing on the Illumina platform, although other methods are available.

As a relatively new field, it is key to understand the structure and complexities of scRNA‐seq data, ensuring that appropriate analytical and statistical methods are applied 21. Particularly challenging is the high level of noise 22, which derives primarily from the nature of single‐cell experiments (called ‘technical variation’ and is mainly due to factors such as mRNA capture efficiency and cDNA amplification bias), along with the biological heterogeneity of cells (‘biological variation’). Furthermore, unlike with conventional RNA‐sequencing where experimental biases are well studied 23, 24, there are biases which are still not fully understood in single‐cell experiments, such as ‘dropouts’ due to the low amounts of starting material, leading to false negative expression.

Single‐cell RNA‐sequencing is a lossy technique, and it is not completely understood what causes the different failure modes for samples. Practically, this means the first step after acquiring reads from a scRNA‐seq experiment is to perform quality control. Reads are processed in a similar manner to bulk RNA‐seq, allowing expression quantification. It is important to check the quality of both the raw data (which can be performed using tools developed for bulk RNA‐seq, such as FastQC 25 or Kraken 26), along with the aligned output. Imperative in scRNA‐seq is the cell‐by‐cell quality control 27, 28, ensuring that cells of poor quality are removed from subsequent analysis. Many metrics can be used to measure cell quality, such as the number of reads or genes detected, the proportion of reads mapping to mitochondrial genes (which may signify leaking of cytoplasmic RNA or cells undergoing apoptosis), or the proportion of reads mapping to externally spiked‐in RNA molecules if used in the experiment 29.

Depending on the analysis task, appropriate normalization of the data is needed. Several normalization methods have been developed, many of which adjust for differences in sequencing depth and/or make use of spike‐in molecules and/or unique molecular identifiers (UMIs) when available (reviewed in detail in 30).

**Figure 1:**
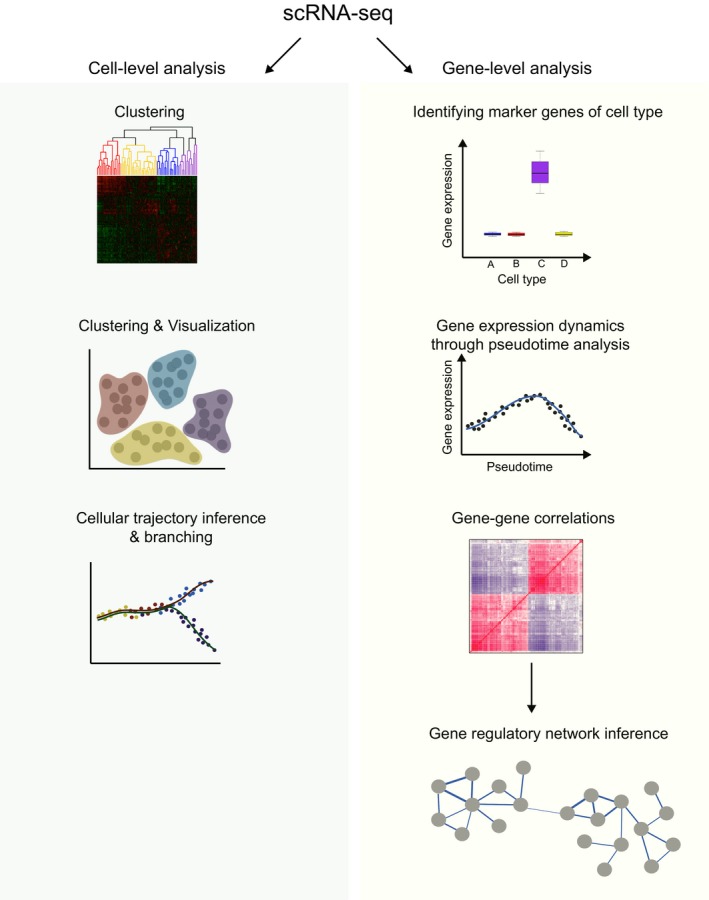
Overview of analysis methods for the interpretation of scRNA‐seq data.

Once cleaned data are obtained, there are many routes of analysis depending on the biological question under investigation (Fig. 1). In this review, we will consider these analysis from two viewpoints: cell‐level approaches, such as the grouping of cells and trajectory ordering, along with gene‐level investigations, such as gene variability and noise, coexpression and identification of differentially expressed genes.

## Cell‐level analysis

### Visualizing and clustering cells

The cataloguing and classification of cells is a long‐standing biological challenge. Traditionally, cell types were determined morphologically or based on molecular cell surface markers. However, with the availability of genome‐wide expression data, the possibility of transcriptome‐based analysis of cell similarity provides an alternative indicator of cell type.

The first step in understanding the distribution of cells is often to apply dimensionality reduction techniques: this represents the thousands of dimensions (genes) found in scRNA‐sequencing data with a much smaller number, attempting to maintain a representation of some variation in interest. Furthermore, by considering only a two or three dimensional space, visualization provides a mean to qualitatively explore the data. There are hundreds of dimensionality reduction methods available (Table 1), which the researcher can elect to apply either to all observed genes or a selected subset of genes of interest. The most widespread is Principal Component Analysis (PCA) 31, where weighted sums of dimensions represent the data. The dimensions for each sample are known as principal components. These dimensions explain decreasing amounts of variation in the original data, with the first principal component capturing as much of the variance as possible. PCA is a simple special case of linear factor analysis. Another commonly applied method is t‐SNE (t‐Distributed Stochastic Neighbour Embedding) 32, a nonlinear visualization technique which considers local distances between data points (cells) by combining dimensionality reduction with random walks on the nearest neighbour network with the goal of separating far‐apart clusters, while also ensuring all data points can be seen by eye to allow for comparisons of cluster size. This is a variation of Multidimensional Scaling (MDS), where PCA is applied on pairwise Euclidean distances to preserve pairwise distances in a low‐dimensional space.

**Table 1:** Tools for the visualization and clustering of cells

Dimensionality reduction and clustering of cells			
Method	Description	Input	Availability
PCA	Linear dimensionality reduction, producing a set of uncorrelated components, explaining decreasing amounts of variation in the data.	Expression table	31
t‐SNE	Nonlinear dimensionality reduction: t‐distributed Stochastic Neighbour Embedding.	Expression table	32
ZIFA	A linear dimensionality reduction technique, using the factor analysis framework, that explicitly models dropout characteristics.	Log‐transformed count values	https://github.com/epierson9/ZIFA 33
Destiny	A fast implementation of diffusion maps for R.	Expression matrix (with a suggested variance stabilized transformation, for example, square root).	http://bioconductor.org/packages/release/bioc/html/destiny.html 34
SNN‐cliq	Graph‐theory‐based algorithm; uses shared nearest neighbour (SNN) graph based upon a subset of genes. The number of clusters is automatically chosen.	Log‐transformation of normalized expression (e.g. RPKM)	http://bioinfo.uncc.edu/SNNCliq/ 35
RaceID	Iterative K‐means clustering of a Pearson correlation matrix, with number of clusters chosen using the gap statistic.	Raw gene expression matrix	https://github.com/dgrun/RaceID 14
SC3	Distance is calculated first, followed by k‐means clustering. Instead of optimizing parameters (e.g. distance metric, matrix transformation), SC3 combines several clustering outcomes and outputs an averaged result.	Normalized expression values	https://bioconductor.org/packages/release/bioc/html/SC3.html 36
SIMLR	Learns a similarity measure from scRNA‐seq data to perform dimensionality reduction, clustering and visualization.	Raw gene expression estimates and number of cell population.	https://bioconductor.org/packages/release/bioc/html/SIMLR.html 37

While powerful, and popular, these techniques can be heavily affected by the problematic abundance of zeroes in single‐cell data; an issue which several methods account for. ZIFA (zero‐inflated factor analysis) 33 extends the linear factor analysis framework, (based on correlations in the data rather than covariances), accounting for dropout characteristics in the data. The R‐package Destiny provides an alternative, nonlinear method using diffusion maps 38: distance between cells reflects the transition probability based on several paths of random walks between the cells. This assumes a smooth nature of the data, and also includes imputation of dropouts.

Unsupervised clustering techniques provide a mechanism to group cells by similarity. While this unbiased approach has benefits, the small number of samples and absence of a way to validate if groupings are ‘real’ poses a problem, along with prior information on the number or type of groups. The features of single‐cell data discussed above, such as dropouts, biases and noise, also add to the difficulty of accurate clustering. Despite these problems, several tools have been developed for use with scRNA‐seq, along with traditional methods such as hierarchical clustering 39. SNN‐Cliq 35 achieves clustering by considering similarity calculated using a graph‐based approach in which a shared nearest neighbour (SNN) network is constructed using rankings of similarities based on expression levels; dense clusters of nodes (cells) are then found. RaceID 14, while also using similarity in expression between cells (based on Pearson correlation), utilizes a different approach: k‐means clustering. In k‐means clustering each sample is associated with one of k prototypes, so that the total squared distance (inverse of similarity) from samples to prototypes is minimal. After the initial step, RaceID uses an outlier detection algorithm and identifies cells which do not fit the model accounting for technical and biological noise. This has been used in the detection of rare cell populations. Another k‐means‐based tool, Single Cell Consensus Clustering (SC3) 36, uses consensus clustering 40, an ensemble strategy, to average over parameter choices in an attempt to make cluster assignments more robust. A recent method, SIMLR 37, uses multiple‐kernel learning to infer similarity in a gene expression matrix with a given number of cell populations. As multiple kernels are used, it is possible to learn a distance measurement between cells that is specific to the statistical properties of the scRNA‐seq set under investigation.

### Cellular trajectory inference and branching analysis

Trajectory analysis is a strictly simpler version of dimensionality reduction, where the assumption is that a 1‐dimensional ‘time’ can describe the high‐dimensional expression values. The theory is that during a biological process, changes will happen gradually, so biological observations can be ordered compared to each other in terms of pairwise similarity. While clustering techniques have been used to define discrete population and states for a long time, trajectory inference is younger in the field of scRNA‐seq.

One of the initial methods for so called Pseudotime analysis of single cells was Monocle 41, which used a minimum spanning tree (MST) strategy to order cells by the distance to a start cell, based on a technique for putting microarray samples on a trajectory 42. In the updated versions of Monocle, the MST strategy has been replaced by a more sophisticated tree‐embedding strategy 43, 44. Monocle is a comprehensive R‐package for single‐cell analysis with functionality for normalization, clustering and differential expression analysis, but the main feature is the pseudotime inference.

Recently, diffusion pseudotime (dpt) has been developed 12. In this technique, geodesic pairwise distances between samples on the data manifold are approximated using a diffusion map representation. Trajectory is then defined as the distance from a start cell along these distances. A different strategy for trajectory inference is to consider a generative model for the data, treating ‘time‐points’ as hidden (or latent). This leads to the probabilistic interpretation of PCA, which in turn leads to factor analysis and ZIFA. Here, the expression of each gene can be described as a linear function of an unknown ‘time’.

Nonlinearity in the data, as described in 41 precludes PCA from being an effective technique for this task. The Gaussian Process Latent Variable Model (GPLVM) allows gene expression to follow any smooth (nonlinear) function over time 45. While more computationally demanding than linear versions, this allows cells to be put in the most likely ordering 45, 46. This means that the most number of genes exhibit smooth expression curves with as little noise as possible. Being a probabilistic model, the benefits are that uninteresting structure in the data can be accounted for directly, such as batch effects or technical factors. It is also possible to incorporate more information about your experimental design through priors 47. There are many implementations of this method. For Python, there is GPy and GPFlow, and for r there are the delorean (https://github.com/JohnReid/DeLorean) and pseudogp (https://github.com/kieranrcampbell/pseudogp) packages.

The Ouija method 48 takes a different approach to pseudotime in a couple of ways. Firstly, it defines a generative model for gene expression in scRNA‐seq data based on ZIFA, to deal with the most common types of measurement noise. Secondly, it is based on the assumption that a small number of switch‐like markers for a biological process of interest are known. The cells are then ordered according to the most likely ordering to confer with the switching genes. Ouija is available as an r‐package on bioconductor and is compatible with the popular scater package 27.

A unique problem in single‐cell developmental data is that a set of progenitor cells can develop into multiple distinct cell types. This means the cells will not follow a single trajectory in the high‐dimensional space. A couple of heuristics have been published: in Wishbone 49, cells are clustered by the pairwise detour distance relative to a reference cell, using geodesic distance. This method is reported to be correctly recovering the known stages and bifurcation point of T‐cell development in mouse. Another method, that has been introduced by Haghverdi *et al*. 12, measures transition between cells using a random‐walk‐based distance.

More principled model based approaches have been presented with SCUBA, which considers transition of cells clusters over time 50. As well as with GPfates/OMGP 47, where multiple smooth trajectories are explicitly modelled. After inference, each cell gets assigned a posterior probability of having been sampled from a particular trajectory. This method has been shown to be efficient in reconstructing the developmental trajectories of Th1 and Tfh cell populations during Plasmodium infection in mice (Table 2).

**Table 2:** Tools for the ordering of cells & bifurcation/branch identification

Method	Description	Input	Availability
Pseudo‐temporal ordering of cells			
PQ‐trees	Samples are ordered by a minimum spanning tree of data, using a PQ‐tree construction.	Expression table	42
Monocle2	A principal graph is embedded in the transcriptome space, distance along the graph from a start cell defines pseudotime.	Expression table, Batch effect formula, gene list (can be found through DE), dimensionality reduction options (method, number of dimensions)	Bioconductor package ‘monocle’ 3
Wishbone	Diffusion maps on reduced k‐NN graph (using waypoints).	Expression table, Start cell, number of waypoints, number of nearest neighbours k.	Python: https://github.com/ManuSetty/wishbone (MATLAB version only supports cytometry data) 49
Wanderlust	Heuristic k‐NN graph geodesic distance	Expression table	In CYT: https://www.c2b2.columbia.edu/danapeerlab/html/cyt-download.html 11
DPT	Diffusion components are averaged for each sample based on spectral embedding, and used as a distance between samples.	Expression table, variance of Gaussian kernel, Start cell	For R and Matlab: http://www.helmholtz-muenchen.de/icb/research/groups/machine-learning/projects/dpt/index.html For Python: https://github.com/Teichlab/scrnatb 12
GPLVM	Assume genes follow any smooth functions and infer time as latent parameter	Expression table or dimensionality reduction, covariance function, optional priors, Optional covariance function hyper parameters.	GPy GPFlow DeLorean pseudogp 45, 46)
Ouija	Provided a small number of genes sigmoidal over trajectory, treat time as latent variable.	Expression table, list of assumed switch‐like genes, optional priors of switching time and direction.	Bioconductor package ‘ouija’. 48
Branching analysis			
Wishbone	Two branches are detected by clustering detours between cells relative to a starting cells in terms of pseudotime.	Expression table	https://github.com/ManuSetty/wishbone 49
Anticorrelation clustering	Branch points are identified when anticorrelated distances (relative to a start cell) become correlated. After this, cells can be segmented to belong to either of the two branches, or the trunk.	Expression table	12
OMGP/GPfates	Model data as a mixture of continuous processes. Each cell obtains a posterior probability of being generated by each of the branches.	Expression table	https://github.com/SheffieldML/GPclust 47
Monocle	The principal graph fitted to the expression data explicitly has the concept of branches, which cells are assigned to.	Expression table, gene list	5
Mpath	Finding Minimum Spanning Tree in neighbourhood graph of landmarks.	Expression table	51

## Gene‐level analyses

### Unwanted factor removal

Uninteresting, largely technical variation can be observed in both bulk RNA‐seq and scRNA‐seq experiments. This variation is usually correlated with some common experimental factor, such as room temperature or stock of reagents. This form of variation is known as batch effects. It is possible to handle batch effects by having a carefully balanced experimental design, such as uniformly distributing replicate conditions across batches. For statistical analysis and inference, if the samples are spread over multiple batches, this information can directly be accounted for 52, 53. Additionally, several statistical methods have been developed to adjust for batch effects 54, 55. One example is ComBat, which removes known batch effects using a linear model of expression from batches where variance is based on an empirical Bayesian framework 54.

Technical variation in scRNA‐seq experiments could be mainly due to mRNA capture efficiency, cDNA amplification bias and the rate cDNAs in a library are sequenced. To estimate technical variation, several methods use spike‐in molecules, which are added with each cell in the same quantity. Risso *et al*. have developed a sleuth of strategies called RUVSeq that either performs factor analysis on a set of control genes such as ERCC spike‐ins or samples within replicate libraries to identify technical factors which can be adjusted for 56. Similar strategies have also been made by others 57, 58, 59.

Grün *et al*. 60 have estimated technical noise in data by fitting a model that incorporates sampling noise and global sample‐to‐sample variability in sequencing efficiency. Subtracting technical noise from total noise has led to inferring the biological noise component, which has been shown to be consistent with single‐molecule FISH, a highly sensitive imaging‐based method for transcript counting 61. An accurate noise model is needed in statistical analysis tasks to avoid overfitting.

A substantial amount of variation also results from differences in cell size or cell cycle stage of each cell. To adjust for cell cycle effects, Buettner *et al*. 62 have developed single‐cell latent variable model (scLVM), which is a two‐step approach that reconstructs cell cycle state before using this information to obtain adjusted gene expression levels by linear regression. They have also shown that removing cell cycle effects in T cells reveals subpopulations associated with T‐cell differentiation 62. This implies the importance of dissecting biological variation into interesting and uninteresting parts in correctly characterizing subpopulations.

### Identification of highly variable genes

Several methods have been developed to identify genes that show high biological variability (Table 3). Brennecke *et al*. 22 have first estimated technical noise using spike‐in molecules, and modelled the mean–variance relationship to identify highly variable genes. Kim *et al*. 7 have presented a statistical framework to decompose the total variance into the technical and biological variance based on a generative model, which would help in identifying variable genes. Another method, BASiCS, uses a Bayesian model which jointly models spike‐ins and endogenous genes and provides posterior distributions for the extent of biological variability 63.

### Identification of differentially expressed genes and marker genes

Identification of differentially expressed genes and marker genes of subpopulations is a simple yet important analysis in scRNA‐seq studies. Although originally developed for bulk RNA‐seq experiments, methods such as DESeq2 64 and EdgeR 65 are also widely used in scRNA‐seq experiments. DESeq2 identifies differentially expressed genes by fitting a GLM for each gene, uses shrinkage estimation to stabilize variance and fold changes, and applies a Wald or likelihood ratio (LR) test for significance testing 64. EdgeR fits a GLM with negative binomial (NB) noise for each gene, estimates dispersions by conditional maximum likelihood, and identifies differential expression using an exact test adapted for overdispersed data 65. Monocle also fits a GLM, but dispersion is estimated directly from the data for each gene, since most single‐cell studies have enough samples to allow this 41. For relative abundance data, dropouts are handled by using a tobit noise model, while using a NB noise model with imputed dropouts for count data.

One of the recent methods developed for scRNA‐seq experiments, called MAST, uses a two‐part generalized linear model that is adjusted for cellular detection rate (dropouts) 66. Another method, M3Drop, applies Michaelis–Menten modelling of dropouts in scRNA‐seq, that is used to identify genes differentially dropped out 67. SCDE is a bayesian method to compare two groups of single cells, taking into account variability in scRNAseq data due to dropout and amplification biases and uses a two‐component mixture for testing for differences in expression between conditions 68. Another method, SINCERA, identifies differentially expressed genes based on simple statistical tests such as Wilcoxon rank sum and *t*‐tests 69. In comparison to these methods, a more recent method, scDD, identifies genes where the overall distribution of values has changed between conditions. This answers a different question which might be of interest in scRNA‐seq experiments 70. Using a Bayesian modelling framework, scDD classifies each gene into one of the four types of changes across two biological conditions: shifts in unimodal distribution, differences in the number of modes, differences in the proportion of cells within modes, or both differences in the number of modes and shifts in unimodal distribution 70.

### Gene‐centric expression dynamics through pseudotime analysis

Using an inferred trajectory as described above, samples can be analysed using a continuous time covariate instead of a few discrete time‐points. This enables the use of more sophisticated time series‐based analysis techniques for modelling gene expression dynamics, and allows us to ask more complex questions from the data.

The popular scRNA‐seq package Monocle provides a wrapper for the VGAM linear modelling package to investigate how expression changes over the trajectory. Splines are used to model expression dependence on pseudotime to allow nonlinear trends. The VGAM package allows for more than just expression levels to be modelled by the splines: with appropriate link functions, allelic expression balance or isoform usage can be modelled 3. Splines require several parameters to be chosen however, and the choices greatly affect the results. A nonparametric nonlinear alternative to spline regression is Gaussian Process regression, which can be used in a likelihood ratio‐based fashion to identify genes which are dependent on pseudotime 45, 71.

Often, we want to ask particular questions from the data, in which case parametric models are useful. In the SwitchDE method, genes which sequentially switched on or off can be identified, along with a parameter letting you learn when the switch happens 72. Similarly, an assumption can be that genes be described as a transient pulse over the pseudotime. The package ImpulseDE identifies such genes, while providing parameters for when in pseudotime the pulse occurs 73.

### Correlation analysis and network inference

One important application of scRNA‐seq studies is the identification of coregulated modules of genes and gene‐regulatory networks constructed using gene‐to‐gene expression correlations. Here, genes with highly correlated expression levels across cells are assumed to be coregulated. Using single‐cell transcriptomic data of Th2 cells, Mahata *et al*. 74 demonstrated how gene–gene correlations can be used to reveal novel mechanistic insights; they have applied correlation analysis between steroidogenic enzyme *Cyp11a1* and cell surface genes and identified *Ly6c1/2* as a marker of the steroid‐producing cell population in mouse.

One method to elucidate regulatory interactions in bulk RNA‐seq studies is called the weighted gene coexpression network analysis (WGCNA) 75. In such a network, nodes represent genes and edges represent coexpression as defined by correlation and relative interconnectedness. The method has also been applied in a scRNA‐seq study where the authors have identified a number of functional modules of coexpressed genes that can describe each embryonic developmental stage in mouse 76.

Although these methods are useful, the inferred networks are undirected; that is, they do not provide direct regulatory relationships among genes. To reveal which gene is upstream/downstream in the regulatory cascade, perturbation experiments (such as knockdown of a gene of interest) are typically required (Table 3).

**Table 3:** Tools for gene‐level analysis

Identification of differentially expressed genes			
Method	Description	Input	Availability
Designed specifically for single cell RNA‐seq data			
SCDE	Bayesian method to compare two groups of single cells, taking into account variability in scRNAseq data due to dropout and amplification biases.	Raw gene expression counts	http://hms-dbmi.github.io/scde/ 68
MAST	Uses two‐part generalized linear model that is adjusted for cellular detection rate.	Normalized gene expression values	https://github.com/RGLab/MAST 66
M3Drop	Applies Michaelis‐Menten modelling of dropouts to identify differential expression.	Raw gene expression counts	https://github.com/tallulandrews/M3Drop 67
scDD	A Bayesian modelling framework to identify genes that are differentially expressed and/or show a differential number of modes or differential proportion of cells within modes.	Normalized and log‐scaled gene expression values	https://github.com/kdkorthauer/scDD 70
SINCERA	Identifies DE genes based on simple statistical tests such as Wilcoxon rank sum and *t*‐tests.	Raw gene expression values	https://research.cchmc.org/pbge/sincera.html 69
Designed originally for bulk RNA‐seq data			
DESeq2	Fits a GLM for each gene, uses shrinkage estimation for dispersions and fold changes, applies a Wald or LR test for significance testing.	Raw gene expression counts	https://bioconductor.org/packages/release/bioc/html/DESeq2.html 64
EdgeR	Fits a negative binomial distribution for each gene, estimates dispersions by conditional maximum likelihood, identifies differential expression using an exact test adapted for overdispersed data. Supports arbitrary linear models.	Raw gene expression counts	http://bioconductor.org/packages/release/bioc/html/edgeR.html 65
Identification of highly variable genes			
Brennecke *et al*.	Biological variability of genes is inferred after quantifying the technical noise based on the square of coefficient of variation (CV^2^) of the spike‐in molecules.	Raw expression counts for both spike‐ins and endogenous genes	22
Kim *et al*.	Presents a statistical framework to decompose the total variance into the technical and biological variance based on a generative model.	Raw expression counts for both spike‐ins and endogenous genes	7
BASiCS	Uses a Bayesian approach that jointly models spike‐ins and endogenous genes. Posterior probabilities associated to highly (or lowly) variable genes are provided.	Raw expression counts for both spike‐ins and endogenous genes	https://github.com/catavallejos/BASiCS 63
Unwanted factor removal			
scLVM	Uses a Gaussian Process Latent variable model to dissect observed heterogeneity into different sources allowing removal of confounding factor of variation such as cell cycle‐induced variations.	Raw gene expression counts and a set of genes associated with the latent factor	https://github.com/PMBio/scLVM 62
Combat	Removes known batch effects based on an empirical Bayesian framework.	Normalized and log‐scaled gene expression counts and batch information	https://github.com/brentp/combat.py/blob/master/R-combat.R 57
OEFinder	Identifies potential artefacts (ordering effects) generated by the Fluidigm C1 platform using orthogonal polynomial regression.	A set of genes (and *P*‐values) that are affected by the artefact	https://github.com/lengning/OEFinder 77
RUVSeq	Adjusts for nuisance technical effects by performing factor analysis on a set of control genes such as spike‐ins or samples such as replicate libraries.	Raw gene expression counts and a set of control genes, spike‐ins or replicate libraries	https://github.com/drisso/RUVSeq 56
Pseudotime Analysis			
Monocle	Spline regression using VGAM	Expression table, gene list	5
SwitchDE	Find genes which are explained as sigmoid curves over pseudotime.	Expression table	Bioconductor package ‘switchde’ 72
ImpulseDE	Find genes which follow an impulse model.	Expression table	Bioconductor package ‘impulsede’ 73
GP Regression	Find genes which follow any non‐linear smooth function.	Expression table	GPy GPFlow Many others

## Conclusions and perspectives

While many tools have been developed to take into account key features of single‐cell RNA‐sequencing data, there is still a way to go. The community will work towards refining existing methods to deal with the complexities of the data, such as the large amount of noise and high level of dropouts. In addition, we are facing issues of scalability due to the increase in experimental throughput, which will also need to be addressed, along with adaptation for changes in experimental protocols. An example of this is the ability to measure gene expression in single cells spatially.

Another major advance will be the combination of other ‐omics techniques, such as the study of methylation 78 and chromatin accessibility 79 in single cells, leading to the same increase in resolution and potential to tackle novel questions as scRNA‐seq. The ability to capture two levels of information within the same cell will hold great power in understanding regulation and functionality at the single‐cell level. This has already been shown with the combination of whole‐genome sequencing and transcriptomics 80, 81 and bisulphite‐sequencing and transcriptomics 82, 83.

Although technical and experimental advances will continue to expand the horizon of research within the single‐cell field, the application to an increasing range of biological areas holds exciting prospects. There has already been significant research into fields in which heterogeneity is well known, such as development 84, 85, immunology 86, 87 and cancer 88, 89. However, the increase in throughput will allow larger investigations. The ability to profile thousands of cells opens the scRNA‐seq field to possibilities such as examining the role of human genetics: how do differences in single‐cell heterogeneity depend on the genetic background of the individual? Furthermore, we are now on the verge of defining all cell types and subpopulations organism wide – creating a ‘Human Cell Atlas’ (www.humancellatlas.org). A thorough description of human cell populations has huge potential to help in understanding disease, and may in future play an important role in clinical diagnosis and treatment.

As the single‐cell field, and the data generation that accompanies it, continues to expand at an incredible rate, it is imperative to develop tools and statistical methods to analyse the data in the best possible way, extracting significant and insightful biological meaning.

## Author contributions

All authors read and approved the final manuscript.
